# The Southern Dental Association

**Published:** 1878-09

**Authors:** 


### ARTICLE V.

### *The Southern Dental Association.*

At a united meeting of the Southern Dental Association and the American Dental Convention, held at Oakland, Md., August 14th, 1877. Dr. W. H. Atkinson, the President of the American Dental Convention, in his opening address, informed the delegates present, that a Committee of three had been appointed by the American Dental Association at its last meeting, to which seven more members had been subsequently added, for the purpose of reporting a plan of organizing the Association into permanent sections, and that to this same Committee had been referred the subject of the formation of a National Representative Association of the Dentists of the whole country. Dr. Atkinson, also, in the same address remarked that he hoped that this Committee would so work as to accomplish what was intended years ago, that is, to have an organization made up of representatives from all State Societies.

The Southern Dental Association at this Oakland meeting, was presided over by the 1st Vice President, Dr. A. C. Ford, of Ga., the President, Dr. S. J. Cobb, of Tenn., being detained at home, on account of illness.

Acting upon the information thus promulgated by Dr. Atkinson, the American Dental Convention before the close of the meeting, appointed a Committee of ten to meet at Niagara Falls, on the day before the meeting of the American Dental Association, in connection with the Committee of the latter body, and also a Committee to be appointed by the President of the Southern Dental Association. Dr. Cobb, when notified of this action, at once appointed a Committee to represent the Southern Dental Association, and actively engaged in efforts to bring about the formation of a National Association. He also assumed the responsibility, after consulting the prominent members of his Association, of changing the place of the next meeting from Atlanta, to Niagara Falls.

Accordingly, the Committees of the Southern Dental Association and American Dental Convention met at Niagara Falls, on Monday, August 5th, 1878, expecting to find there a similar Committee from the American Dental Association.

At the first meeting of these Committees, it was apparent that the Committee on Sections, of the American Dental Association, had no power whatever to even consider the subject of the formation of a National Association, and a subsequent meeting more largely attended than the first, revealed the fact that the American Dental Association had not the slightest idea of giving up their organization for the purpose of uniting with the two other Associations in the formation of a National one. The majority of the members of the Committees from the Southern Dental Associaton and the American Dental Convention, were greatly disappointed at this result of an effort they had confidently magined would lead to the organization of one National Association, and the mistake of supposing that the American

Association was favorable to such a project, was due to the statement made by Dr. Atkinson, the President of the American Dental Convention, in his opening address at the Oakland meeting.

The following is the form of notice sent to the members of the two Committees by their respective Chairmen:

"Dear Sir.—The members of the Committees appointed by the American Dental Convention and the Southern Dental Association, for the purpose of perfecting their report on the subject of the formation of a National Dental Organization, will please meet punctually at the "Cataract House," Niagara Falls, on Monday, August 5th, at 9 o'clock, A. M., where, it is confidently expected, a Committee from the American Dental Association will be present with us. The importance of this movement makes it imperative that the above Committees shall be well represented, and we therefore cannot too strongly urge your punctual attendance."

The members of the Committee on Sections, of the American Dental Association, were very confident that no power had been given them to act in this matter, and in proof of this, referred to the printed proceedings of the Chicago meeting, wherein no mention whatever of this matter occurs.

On Thursday morning, August 8th, the Southern Dental Association met at the "Cataract House," Niagara, Dr. S. J. Cobb, presiding, and Dr. E. S. Chisholm, Rec. Secretary. Some thirty members of this Association were at the Falls, and after transacting its regular business, and a free discussion concerning the failure of the effort to organize a National Dental Association, the Southern Dental Association proceeded to elect the officers for the ensuing year, as follows:

*President.*—Prof. F. J. S. Gorgas, Baltimore, Md.

1*st Vice President.*—Dr. L. D. Carpenter, Atlanta, Ga.

2*nd Vice President.*—Dr. J. R. Walker, New Orleans, La.

*3rd Vice President.*—Dr. John G. Wayt, Richmond, Va.
*Corresponding Secretary.*—Dr. A. C. Ford, Atlanta, Ga.
*Recording Sec'y.*—Dr. E. S. Chisholm, Tuscaloosa, Ala.
*Treasurer.*—Dr. H. A. Lowrance, Athens, Ga.
*Executive Committee.*—Dr. W. C. Wardlaw, Augusta, Ga., Dr. G. W. Winkler, Augusta, Ga., and Dr. G. W. H. Whittaker, Sandersville, Ga.

Augusta, Georgia, was selected as the place for the next annual meeting, which will be held on the second Tuesday in July, 1879. As the Georgia State Dental Association meets in Augusta, at the same time, and also the Associations of South and North Carolina, near this time, it is confidently expected that the next annual meeting of the Southern Dental Association will be the largest known in its history.

